# Sex Chromosome Dosage Compensation in Insects

**DOI:** 10.3390/insects16020160

**Published:** 2025-02-04

**Authors:** Xingcheng Xie, Yakun Zhang, Heyuan Peng, Zhongyuan Deng

**Affiliations:** 1 School of Agricultural Sciences, Zhengzhou University, Zhengzhou 450001, China; xingchengxie@126.com (X.X.); pengheyuan123@outlook.com (H.P.); 2 State Key Laboratory for Biology of Plant Diseases and Insect Pests, Institute of Plant Protection, Chinese Academy of Agricultural Sciences, Beijing 100193, China; zhangyakun7@126.com

**Keywords:** dosage compensation, dosage compensation complex, sex determination, sex chromosome, gene expression

## Abstract

Dosage compensation (DC) is of crucial importance in balancing the sex-linked gene expression between males and females. It serves to guarantee that the proteins or other enzymatic products encoded by the sex chromosome exhibit quantitative parity between the two genders. During the evolutionary process of achieving dose compensation, insects have developed a wide variety of mechanisms. There exist two primary modes of dosage compensation mechanisms, including the up-regulation of heterogametic sex chromosomes in the heterogamety and down-regulation of homogametic sex chromosomes in the homogamety. Although extensive investigations have been conducted on dosage compensation in model insects, many questions still remain unresolved. Meanwhile, research on non-model insects is attracting increasing attention. This paper systematically summarizes the current advances in the field of insect dosage compensation with respect to its types and mechanisms. The principal insects involved in this study include the *Drosophila melanogaster*, *Tribolium castaneum*, *Bombyx mori*, and other lepidopteran insects. This paper analyzes the controversial issues about insect dosage compensation and also provides prospects for future research.

## Simple Summary

The dosage compensation effect is one of the most common mechanisms by which the organisms with sexual dimorphism balance the genes expression. In insects, there mainly exist four types of sex determination, including the male heterogamety XY/X0 and the female heterogamety WZ/Z0. Although there has been a wide variety of studies on the dosage compensation effect, there is still a lack of systematic review. Moreover, some studies are even controversial. Therefore, it is essential to carry out a comprehensive and systematic analysis of previous studies. In this review, we summarized the research progress, analyzed the existing problems, and explored future study prospects. The purpose of this review is to provide references for the future work on sex chromosome dosage compensation in insects.

## 1. Introduction

There are numerous methods of sex determination in organisms, and diverse species of organisms have distinguishable types of sex determination [1]. Sex determination refers to the developmental program that determines whether an embryo will follow either the male or the female pathway [2]. In individuals that reproduce sexually, sex determination is based on the homotypic (such as XX) or heterotypic (such as XY) sex chromosomes [3]. For the XY-type sex determination observed in mammals and fruit flies, males have XY sex chromosomes, whereas females have XX sex chromosomes [4]. However, in the WZ-type sex determination system of many lepidopteran insects, birds, fish, reptiles, and amphibians, the ZZ and WZ sex chromosomes determine males and females, respectively [5]. In organisms in which gender is determined by one sex being homomorphic and the other heteromorphic with regard to the sex chromosomes, a process has evolved to equalize the output of products encoded by the genes located on these chromosomes [6]. This process is known as dosage compensation, and the phenomenon associated with this process is called the dosage compensation effect [7,8]. Dosage compensation is extremely common in bisexual eukaryotes. In 1932, Muller discovered a regulatory mechanism that balances the expression of X-linked gene between male (XY) and female (XX) in *Drosophila melanogaster*, after studying the eye pigment level of individuals with X-linked gene deletion mutations [9]. This mechanism was named as the “dosage compensation mechanism” [9]. Subsequently, achievements were obtained in mammals and *Caenorhabditis elegans*, which revealed three possible dosage compensation mechanisms [10,11]: doubling the expression of a single X chromosome in males (*D. melanogaster*), inactivating one of the female X chromosomes (mammals), and halving the expression of the female’s two X chromosomes (*C. elegans*). The evolutionary strategies of dosage compensation across species, such as *D. melanogaster*, mammals, and *C. elegans*, have also been compared [12].

The acquisition of male-specific lethal (MSL) mechanisms in *Drosophila* and the successful identification of the dosage-compensating MSL complex have outlined the molecular basis for the mechanism of dosage compensation in *Drosophila* [13]. The MSL complex is a nucleoprotein RNA-containing protein complex that consists of at least five specific proteins and two non-coding RNAs. It is the core of the *Drosophila* dosage compensation machinery [14]. The functional factors, patterns, and mechanisms of *Drosophila* dosage compensation are thoroughly understood.

Unlike *Drosophila*, mammals obtain the dosage compensation by inactivating one of the X chromosomes in females. Although the composition of the mammalian MSL complex has been determined, its function remains largely unknown [15,16]. So far, research on the dosage compensation types and mechanisms of insects remains in the initial stage. There still exist some controversies regarding the dosage compensation types of insects. However, significant progress has been made in the research on model insects. Drawing on the literature reports and experimental studies, this paper undertakes a review of research on dosage compensation types and mechanisms. It also discusses the causes and issues surrounding the controversial dosage compensation. Additionally, it provides prospects for the further study of dosage compensation in insects.

## 2. Sex Determination and Differentiation

The evolution of sex determination in organisms has been widely investigated over the years. However, the origin of sex chromosomes in insects is currently not fully understood. Typically, sex chromosomes are postulated to originate from a pair of ancestral autosomes that have evolved a sex-determining locus [17]. Accompanied by the sex-biased segregation of sex chromosomes, a proto-X or Y chromosome is also defined in female or male heterogametic organisms, respectively [18,19]. In accordance with the diversity and flexibility in the evolutionary process, sex differentiation has become increasingly distinct and complex [20]. For the organisms exhibiting sexual dimorphism, sex determination is a crucial step in developmental differentiation [21]. This process is essential for normal development and survival of individuals, as well as the genetic basis that enables the continuation of species reproduction.

**Figure 1:**
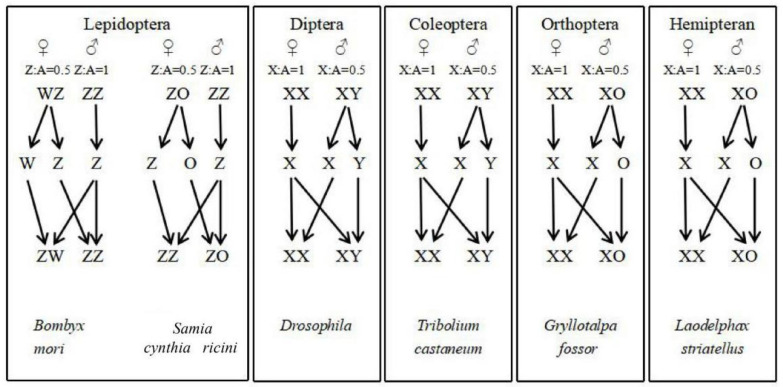
Sex determination types in different insects. The representative insects used for sex determination analysis are *Bombyx mori* [35], *Samia cynthia ricini* [30], *Drosophila* [36], *Tribolium castaneum* [37], *Gryllotalpa fossor* [38], and *Laodelphax striatellus* [39], respectively.

Generally speaking, there exist two main sex determination mechanisms, namely, genetic sex determination (GSD) and environmental sex determination (ESD) [22]. Although sex determination is particularly crucial for the sexual fate of organisms, it remains one of the variable developmental pathways that is influenced by the living environment and chromosomes of the organisms [23]. The sex determination by chromosomes is particularly universal and closely related to the dosage compensation effect. Furthermore, sex chromosome determination mainly presents in the following types [24]: males with the heterogamety XY and X0 and females with the heterogamety WZ and Z0 (Figure 1).

1. WZ-type sex determination: The sex determination of birds, some lepidopteran insects like silkworms and bollworms, as well as crustaceans is of WZ type. WZ type is also common in snakes and some lizards, turtles, amphibians, and fish [25]. This type of female is heterogametes (namely, WZ), while males are homogametes (namely, ZZ) [26]. During the process of gamete formation, females generate female gametes of Z and W types, whereas males only produce male gametes of Z type. When a male gamete Z binds to a female gamete Z, the fertilized egg can develop into a male. If a male gamete Z combines with a female gamete W, the heterozygote continues to grow into a female. Therefore, the ratio of males to females is 1:1 [27].
2. Z0-type sex determination: As a specific type of the WZ sex determination, it is commonly referred to as Z0 sex determination [28,29]. This particular type of sex determination is mainly observed in certain species of butterflies and moths [3]. In these organisms, males have two homomorphic sex chromosomes (ZZ) and only produce one type of gamete, namely, Z [30]. However, females only have an odd sex chromosome (Z) [30]. In this case, due to the absence of maternal W sex chromosome, offspring with ZZ embryos develop into males, while those with Z0 embryos develop into females. For example, a moth of Psychidae, *Talaeporia tubulosa*, employs ambient temperature to govern sex determination in the absence of the W chromosome [31]. During the meiosis process of female individuals, environmental factors may occasionally affect the distribution of Z chromosomes in *T. tubulosa* [31]. At high temperatures, more eggs will carry Z chromosomes, thereby resulting in a higher proportion of males. In contrast, at low temperatures, fewer eggs contain the Z chromosome, which leads to more female offspring [31].
3. XY-type sex determination: It is quite prevalent among animals, encompassing most insects, mollusks, amphibians, and mammals. There exist multiple differences between XY-type and WZ-type sex determination. The female is of the homogametic type (namely, XX), whereas the male is of the heterogametic type (namely, XY). The size and morphology of Y and X chromosomes are different. During meiosis, the female produces only one type of gamete (X), while the male produces two kinds of gametes (X or Y). When the female gamete combines with X sperm, the fertilized egg develops into a female. If it is integrated with Y sperm, the egg develops into a male. Therefore, the sex ratio is generally 1:1 [32].
4. X0-type sex determination: A particular kind of XY sex determination is X0-type sex determination [33], which is analogous to the Z0 type. Insects of this type primarily consist of locusts, crickets, and scorpionflies [33]. The X (or Z) chromosome is generally larger than the Y (or W) chromosome. In nematodes, sex is determined with high fidelity based on the tallying of X-chromosome number in relation to ploidy and the sets of autosomes (referred to as the X:A signal). This process is carried out through a precise strategy. Embryos with ratios of 1X:2A (0.5) or 2X:3A (0.67) develop into fertile males. In contrast, embryos with ratios of 3X:4A (0.75) or 2X:2A (1.0) develop into self-fertile hermaphrodites [34].

In general, the composition of sex chromosomes determines the gene doses on sex chromosomes. Nevertheless, there is no difference in the performance of biological X (or Z)-linked traits among males. In some organisms with heterotypic sex chromosomes, such as birds having WZ sex chromosomes, it is not always necessary to compensate the gene dose. However, the differences in gene sets may affect gene expression from the same copy as in the homogametic sex [40]. There should be a mechanism to maintain the consistent gene expression levels between the two sexes, and this was later confirmed as the dosage compensation.

## 3. The Dosage Compensation Effect

### 3.1. Research on the Dosage Compensation Effect

Sex determination occurs after conception as the result of XX or XY chromosomes pairing [41]. Nevertheless, sex chromosomes have evolved repeatedly. When chromosomes exist in different copy numbers between males and females, they are likely to be subject to different selection pressures compared to the autosomes [42]. Subsequently, this leads to a faster rate of evolution, increased accumulation of sexually antagonistic alleles, and the evolution of dosage compensation [42]. Through the comparison of closely related species, research indicates that heterotypic sex chromosomes evolved from a pair of common chromosomes carrying sex-determining factors [43]. Moreover, the existence of a dosage compensation mechanism was firstly proven at the experimental level in *D. melanogaster*, and the results demonstrated that the amount of H^3^ uracil in a single X chromosome of the male fly involved was equal to the number of H^3^ uracil inserted into the two X chromosomes of the female fly by the method of autoradiography [44]. Therefore, it was concluded that the amount of RNA produced on the X chromosome of males is approximately twice as much as that on a single X chromosome of females. This above-mentioned phenomenon was subsequently confirmed in mammals and nematodes.

The dosage compensation effect of three model organisms, including the nematodes, insects, and mammals, has also been investigated [45]. For these representative species with distant genetic relationships, there are distinct dosage compensation mechanisms. However, most dosage compensation is achieved by regulating the expression of homotypic chromosomal-linked genes [34]. Dosage compensation strategies vary from worms to mammals. Nevertheless, there still remains a regulatory complex that can integrate with the X chromosomes of one sex to regulate the transcription along the entire chromosome. For mammalian females, there are two X chromosomes, but the X inactivation-specific transcript (Xist) functions to inactivate one of the X chromosomes, thereby achieving dosage compensation by imprinted and random X-chromosome inactivation [44,46]. In other words, dosage compensation is obtained through inactivating one X chromosome in females [43,47].

The dosage compensation effect in fruit flies is achieved by increasing the gene transcription amount on the X chromosome in males, so that it is approximately equal to the gene transcription number on the two X chromosomes in females [45,47]. *C. elegans* has male (X0) and facultative (XX) forms. The XX facultative individuals can achieve dosage compensation by halving the transcription efficiency of two active X chromosomes [45,48,49]. Gene expression along X chromosomes is regulated by dosage compensation and, simultaneously, histone modifications as well as chromosome structure are also employed to modulate gene expression across X chromosomes [34].

Studies on aneuploid organisms have demonstrated that the gene dosage effect on gene expression is considered variable because of the dosage changes in chromosome fragments. This can be further classified into two effects. The first effect is the positive gene dosage effect. Namely, the gene expression level shows a positive correlation with its dosage. The other one is the inverse dosage effect, which refers to an inverse relationship between gene expression and the copy number of a trans region. This implies that there is a reduction in gene expression when the dosage of another chromosome fragment is increased. The inverse dosage effect is predominant in aneuploid organisms such as *Arabidopsis*, maize, *Datura*, *Drosophila*, and human beings. Dosage compensation ensures the equal expression of different dosages of X chromosomes between females and males, which is conducted by a two-fold up-regulation of the gene expression on the single X chromosome in males to match the gene expression level of the two chromosomes in females. This phenomenon is a consequence of the simultaneous action of the positive and inverse dosage effects [50].

However, studies on dosage compensation are sometimes conflicting [51]. There may also exist complete, partial, and incomplete dosage compensation mechanisms. In these mechanisms, the up-regulation of sex-related genes occurs on a gene-by-gene basis [52]. Incomplete dosage compensation refers to sex-linked expression in the heterogametic sex that is reduced in comparison to ancestral expression, which leads to an expression imbalance and sex-biased gene expression on the sex chromosome between male and female individuals [53]. Incomplete dosage compensation occurs in XY species, which is much more common than that in WZ species. The dosage compensation pattern of orthopteran insects is similar to that of the mammals. Similar to the XY type, however, orthopteran insects only have one sex chromosome. In Orthoptera, there are two genders: male (X0) and female (XX). A study demonstrated that phosphoglucomutase (PGM) is an X-linked gene in orthopteran insects. PGM inhibits the gene expressions on one X chromosome in female individuals, thereby making the gene dosage of PGM roughly equal between males and females [54]. In addition, some studies have also indicated that the dosage compensation exhibits different patterns depending on different somatic tissues. For instance, the evolution of the X chromosome was characterized in five species of *Timema* stick insects [42]. Through whole-genome sequencing, the type of sex determination was identified as XX:X0 [42]. It was concluded that there was almost complete dosage compensation in somatic tissues (heads and legs), whereas the reproductive tracts were lacking in dosage compensation effect [42]. Furthermore, a complete dosage compensation mechanism was also discovered in a WZ species known as *Manduca sexta*, which is similar to the genome-wide sex gene expression pattern [55]. This finding indicated that the tobacco hornworm balances the key genes in the genome-wide network through full dosage compensation.

In birds, the Z chromosome is partially up-regulated in females, but partially or randomly down-regulated in males. Incomplete dosage compensation in chicken is achieved by up-regulated genes on the Z chromosome in females. The dosage compensation effect of silkworms and Coleopteran insects has also been investigated. For example, *Tribolium castaneum*, the red flour beetle belonging to Coleoptera, shows incomplete dosage compensation [56,57]. However, for the silkworm, which is one of the most deeply studied model insects of Lepidoptera, there are still some controversies regarding its dosage compensation mechanism [58]. Therefore, the dosage compensation mechanism of insects, particularly those with the ZW sex system, needs further comprehensive and more in-depth research.

Generally speaking, the dosage compensation effect occurs only in the sex chromosomes [59]. However, this effect can also be observed in individuals with certain autosomal abnormalities like aneuploidy, as well as in those with only specific autosomal segments [60]. For example, the alcohol dehydrogenase-1 (*ADH1*) gene on the long arm of maize chromosome one (1L) [61] and *α*-glycerophosphate dehydrogenase (*α-GPDH*) genes located on the left arm of *Drosophila* chromosome 2 (2L) [62] both show a dosage compensation effect. However, the dosage compensation mechanism of autosomes need further investigation.

Due to the diversity of sex determination mechanisms in insects, although some insects exhibit strong homology in morphology and biological habits, research on dosage compensation in insects remains challenging and progresses slowly. For some insects, genetic differences can determine the sex of females and males [63]. However, for other insects, environmental conditions may also determine the sex [63]. Even within a single sex chromosome, there is a possibility of the coexistence of two distinct sex chromosome dosage compensation (SCDC) patterns [64]. For instance, in the monarch butterfly, different dosage compensation modes have been observed on the two Z segments, namely, the neo-Z and anc-Z segments [65]. Therefore, so far, only *D*. *melanogaster* among Diptera species has a clear dosage compensation mechanism [66]. Further research on dosage compensation in *Bombyx mori*, *M*. *sexta*, and *T*. *castaneum* is necessary considering that there are many controversies and a lack of sufficient studies on the dosage compensation of these three insects. To better study the dosage compensation of insects, we have summarized the currently known mechanisms of dosage compensation in different insects (Table 1).

**Table 1:** Dosage compensation indifferent insects.

Order	Species	Sex Mechanism (Female/Male)	Sex Chromosome Dosage Compensation Patterns	Reference
Diptera	*Drosophila melanogaste*	XX/XY	Complete	[36,67]
	*Diopsidae*	XX/XY		[68]
	*Lucilia cuprina*	XX/XY		[69]
	*Anopheles gambiae*	XX/XY		[70,71,72]
	*Anopheles stephensi*	XX/XY		[73]
Coleoptera	*Tribolium* *castaneum*	XX/XY	Complete	[37,74]
Strepsiptera	*Xenos vesparum*	XX/XY	Complete	[74]
Hemiptera	*Halyomorpha halys*	XX/XY	Complete	[75]
	*Homalodisca vitripennis*	XX/XY		
	*Oncopeltus fasciatus*	XX/XY		
	*Acyrthosiphon pisum*	XX/X0		[76]
	*Laodelphax striatellus*	XX/X0	Complete (in somatic tissues)	[39]
Phasmida	*Timema*	XX/X0	Complete (in heads and legs)	[42]
Orthoptera	*Gryllotalpa fossor*	XX/X0	Complete	[38,77]
	*Acheta domesticus*	XX/X0		
Lepidoptera	*Bombyx mori*	WZ/ZZ	Complete	[58,78,79,80]
	*Manduca sexta*	WZ/ZZ	Complete	[55]
	*Cydia pomonella*	WZ/ZZ	Complete	[25]
	*Plodia interpunctella*	WZ/ZZ	Incomplete dosage compensation with balance	[81]
	*Heliconius*	WZ/ZZ	Complete	[82]
	*Papilio xuthus*	WZ/ZZ	Complete (excluding sex-biased genes)	[80]
	*Papilio machaon*	WZ/ZZ	Complete (excluding sex-biased genes)	
	*Helicoverpa armigera*	WZ/ZZ	Complete	[83,84]
	*Leptidea sinapis*	WZ/ZZ	Complete	[85]
	*Samia cynthia ricini*	Z0/ZZ	None	[30]
Hymenoptera	*Apis mellifera*	2N/N	Single-locus complementary sex determination	[86]

### 3.2. The Dosage Compensation Mechanism of the Fruit Fly

**Figure 2:**
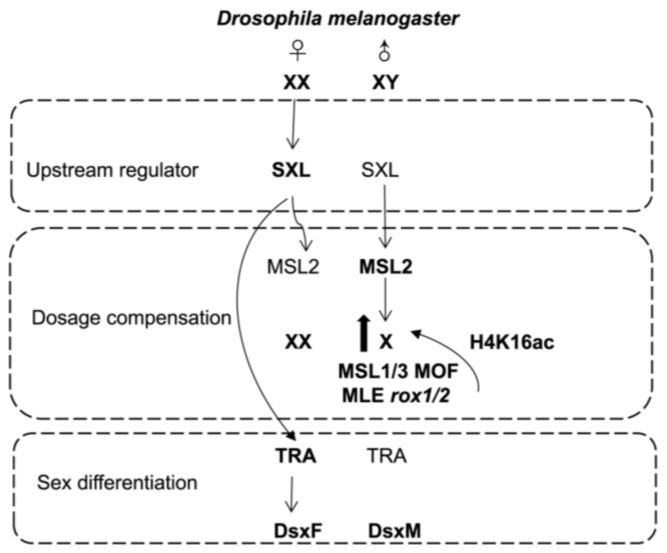
Schematic diagram of the dosage compensation mechanisms involved in *Drosophila* [24,66]. See text for details.

As a representative organism, *Drosophila* achieves dosage compensation by up-regulating the genes on the male X chromosome and doubling the gene expression [87,88]. In somatic cells of *Drosophila*, the dosage compensation mechanism is accomplished when the dosage compensation complex (DCC) directly binds to the X chromosome, acetylates the X chromosome, and causes X-linked genes to be hypertranscribed [89,90]. Several subunits of MSL in DCC are not expressed in male germline cells, but there is still partial dosage compensation [89]. In somatic cells, all DCC condenses and SDC (sex determination and dosage compensation) subunits bind to X chromosomes, followed by starting about the 30-to-40-cell stage of embryogenesis. DCC binding is maintained throughout the adulthood [34]. Currently, this mechanism is the one best understood [24,66] (Figure 2).

#### 3.2.1. Upstream Regulatory Signals of Drosophila Dosage Compensation

*Drosophila* dosage compensation is regulated by the alternative splicing of upstream gene *sex lethal* (*Sxl*), which results in its specific expression in males and females [36,91,92]. In males, the *Sxl* transcript retains exon 3 with a stop codon, which makes the *Sxl* gene translate into a short and non-functional SXL protein. In females, exon 3 is spliced out, and the *Sxl* gene is transcribed completely. The intact open reading frame of the transcript is responsible for encoding a functional SXL protein [93]. The female SXL protein binds to the sites in the 5′UTR and 3′UTR of the *msl2* gene and prevents the translation of *msl2* gene [94]. Males have non-functional SXL protein and allow for the *msl2* gene to translate into active MSL2 protein along with other components, which subsequently initiate the dosage compensation effect [95,96]. In females, the *Sxl* gene regulates the splicing of *doublesex* (*dsx*) genes by activating *transformer* (*tra*) genes through accurate splicing [97]. Subsequently, Tra-2 acts as a cofactor to generate the female isoform DsxF, a female-specific Dsx [98]. However, in males, due to the non-functional SXL protein and the absence of Tra protein, *dsx* pre-mRNA is spliced to generate the male-specific Dsx (DsxM). These two proteins, DsxF and DsxM, induce the sex-specific phenotypic changes [99]. Moreover, the existence or absence of Tra protein is crucial for determining sex differentiation [50].

#### 3.2.2. The *Drosophila* DCC

The components of *Drosophila* MSL complex were identified through screening in the *male-specific lethal* (*msl*) genes. Subsequently, genes carrying this mutation were cloned and characterized [57]. The dosage compensation complex of *Drosophila* is encoded by five genes, namely, *msl1*, *msl2*, *msl3*, *maleless* (*mle*), and *males absent on the first* (*mof*), as well as two non-coding RNAs, namely, *roX1* (*RNA on the X1*) and *roX2* (*RNA on the X2*), which is also known as MSL complex [66,100]. Chromatin proteins, such as JIL-1 tandem kinase, Megator (Mtor), Chromatin-linked adapter for MSL proteins (CLAMP), Topoisomerase II (TopoII), and other proteins, may represent additional components of the MSL complex. These chromatin proteins have been shown to co-immunoprecipitate with the core MSL subunits [89]. The genes of *msl1*, *msl3*, *mof*, *mle*, and non-coding RNA can be expressed in both males and females. However, the *msl2* gene is only expressed in males [101]. These components of the MSL complex collaborate at hundreds of discrete affinity sites on the X chromosome of male *Drosophila* but not those of other chromosomes [50]. The MSL complex is a crucial determinant for the survival of male flies, and the absence of any components is specifically lethal to male flies [50,102,103]. Therefore, it is indicated that the MSL complex is essential for insects in the dosage compensation mechanism [10,104].

The assembly sequence of the MSL complex and the interaction site of each component have invariably been the focal points of researchers. Although the reported sequence of MSL complex assembly may vary slightly (possibly due to the different tissues examined or the different cell lines in cell culture), it is definite that MSL1 and MSL2 function as substrates and provide a platform for the assembly of other proteins and *roX* RNAs. Moreover, the interaction between MSL1 and MSL2 is required for the binding of MSL complex to chromosomal entry sites (CESs) on the X chromosome. The addition of MSL3, MOF, and MLE is essential for the extension of the complete MSL complex. *roX* RNAs also play a key role in the localization of the MSL complex [50].

In general, MSL1 serves as a platform for assembling the MSL complex [95,105]. How does MSL1 bind to MSL2 and initiate the assembly of the MSL complex? Experiments have demonstrated that the amino terminus of the MSL1 protein recognizes the X chromosome [106,107]. At the carboxyl terminus, there exists a relatively conserved region known as the PEHE structure. Because of its characteristic amino acid composition, the PEHE structure functions as the binding region with MSL3 and MOF [108]. MSL1 and MSL2 form a core protein complex, which initiates the assembly of the dosage compensation complex [109]. As a crucial factor in dosage compensation [110], MSL2 is a RING-finger protein with a RING-finger at its amino terminus for binding to MSL1 [111]. Owing to the lack of MSL2 protein, the dosage compensation complex fails to be formed in females of *Drosophila* [112].

However, how do the other components of the complex successively bind to the MSL1/MSL2 platform? The conserved proline domain in MSL2 is *roX* RNA, which is necessary for the efficient incorporation of MSL complex and stabilization of *roX* RNA [113,114]. The condensing characteristic of the roX-MSL2^CTD^ determines the segmentation of the *Drosophila* X chromosome. After specific recruitment by the X chromosome, the C-terminal domain (CTD) of MSL2 is sensitive to *roX* non-coding RNA. Functional analysis of *Drosophila* and mammalian cells reveals that *roX* non-coding RNA and MSL2 CTD assemble into a stable condensation state, and the interaction between them is essential for the dosage compensation in vivo [109]. The conserved cysteine CXC domain of MSL2 enables MSL2 to target the X chromosome instead of the autosomes, and the core protein complex consisting of MSL1 and MSL2 can also bind to the male X chromosome through the CXC domain [115,116]. The CD domain located at the N-terminus of MSL3 is the region that binds to nucleosomes, nucleic acids, and histone H3, whereas the C-terminus is the binding region for two non-coding RNAs [117]. MSL3 is involved in the diffusion of the MSL complex from the binding site on the X chromosome to other sites [118,119]. Inactivation of the MSL3 protein results in the binding of the incomplete MSL1-MSL2 complex to approximately 200 sites on the X chromosome, which are referred to as chromatin entry sites (CESs) or high-affinity sites (HASs) [116].

A histone acetyltransferase MOF is part of the non-specific lethal (NSL) complex. MOF acetylates the sixteenth lysine (H4K16) on male histone H4 by combining with housekeeping genes throughout the whole genome, which neutralizes the positive charge and weakens the inhibition of the internucleosomal structure, thereby enhancing the transcription of the X chromosome [120,121]. Moreover, MOF is the only acetylase that utilizes histone modifications to achieve decondensation of the entire chromatin [122]. Although MLE is an RNA/DNA helicase with an ATPase domain, MLE does not bind to any of these proteins [123,124]. Instead, only RNA via *roX* RNA is assembled into the MSL complex [125]. *roX1* and *roX2* are non-coding RNAs with specific secondary structures on X chromatin [126,127] that primarily bind to MSL complexes and carry out specific mediating functions [128,129]. The low-complexity CTD of MSL2 makes its recruitment to the X chromosome sensitive to *roX* non-coding RNAs. *roX* non-coding RNAs and the CTD of MSL2 form a solidly condensed state, and function analysis further indicates that their interactions are essential for dosage compensation in *Drosophila* and mammalian cells [109]. In addition to these core dosage compensation complexes, there still exist many accessory factors involved in the dosage compensation effect. These factors also facilitate the targeted localization and extension of the MSL complex on the X chromosome [130,131].

The current model, which illustrates the delivery of the MSL complex to nearly all expressed genes on the male X chromosome, mainly consists of two steps [14,132]. Firstly, the MSL complex binds to approximately 200 HAS/CES on the X chromosome [116]. Subsequently, the MSL complex spreads from HAS/CES to the nearby expressed genes due to the association of MSL3 subunit with Lysine 36 of histone H3 (H3K36me3) modification over these genes [133]. There exist one to several copies of a 21-bp GA-rich sequence motif in each HAS/CES [89]. This motif is called the MSL recognition element (MRE) [134]. However, only a small number of all MREs are localized within HAS/CES [89]. The DNA-binding protein CLAMP has the ability to recognize MREs and is beneficial for the recruitment of MSL to HAS/CES. In consideration of the physical interaction between MSL2 and CLAMP, the CXC and CLAMP-binding domains are essential for the MSL complex to target the X chromosome [89]. More precisely, the first zinc-finger C2H2 domain at the N-terminus of CLAMP interacts with the unstructured highly conserved CLAMP-binding domain (within the *Drosophila* genus) of MSL2 [116]. The structural basis for the interaction between the CLAMP and MSL2 proteins is obtained by the methods of NMR techniques and mutagenic screening experiments [116]. Further study shows that the amino acid residues employed for the above interaction are different from those commonly used for DNA recognition [116]. Moreover, CLAMP is capable of opening the chromatin, and its recognition of MREs may enhance the binding of MSL2 [89].

The MSL complex can specifically bind to the X chromosome. How does the MSL complex precisely localize the X chromosome and initiate dosage compensation? The intensity of CES on the X chromosome determines the affinity of the MSL complex for the X chromosome. Some studies have demonstrated that the MSL complex can specifically bind to the X chromosome and enhance its transcription. Furthermore, the MSL complex number is also crucial for accurate localization on the X chromosome. Although low levels of MSL complex can bind to the X chromosome, the binding sites are significantly diminished. In contrast, high levels of MSL complex may lead to abnormal binding to the chromocenter and the fourth chromosome [13,135]. When the number of MSL complex reaches a critical value, the dosage compensation effect occurs. Otherwise, even if a small number of MSL complex bind to the sex chromosome, the gene transcription does not increase [136]. However, it remains unclear how this doubled precision is accomplished. Recent studies have shown that a nuclear pore component, Mtor, determines the appropriate transcription levels from the male X chromosome [104]. Both Mtor and the components of MSL complex are involved in regulating the levels of dosage-compensated expression [104]. The effects of genomic imbalance on MSL complex and sex chromosome aneuploidy have been further investigated. The results indicate that most X chromosome genes still exhibit dosage compensation compared with normal diploid females [50].

Although the dosage compensation mechanisms in *Drosophila* have been widely studied, many questions still remain unanswered. For example, what is the kinetic basis for the binding of the MSL complex to the X chromosome? What is the specific molecular mechanism by which the MSL complex recognizes the X chromosome and targets the target genes? Are there any other DNA sequence elements that promote the assembly or disassembly of the MSL complex? These problems need further exploration by researchers in future work.

### 3.3. The Dosage Compensation Mechanism of the Red Flour Beetle

The red flour beetle, *T. castaneum* (Coleoptera; Tenebrionidae), is a major pest of stored grains and is widely distributed in temperate zones around the word [137]. Nevertheless, the dosage compensation mechanism of *T. castaneum* remains controversial. Prince *et al.* pointed out that the mechanism of the red flour beetle is an incomplete dosage compensation mechanism [37]. In the red flour beetle, the expression of male sex chromosomes consistently increases along with autosomes, whereas the expression of female sex chromosomes also shows an increase [37]. Compared with the DCC of *D. melanogaster*, *T. castaneum* has the *msl2*, *msl3*, *mof*, and *mle* genes, but lacks the *msl1* gene [138]. Analysis of the larval RNAi experiment showed that after the knockdown of the *mof* gene, in contrast to the male lethality observed in *D. melanogaster*, the mortality for both females and males of *T. castaneum* was 100% [138]. Further study also revealed that there was no obvious male-lethal effect for the other three DCC genes, namely, *msl2*, *msl3*, and *mle* [56]. Therefore, these results indicate that there may be no dosage compensation mechanism in *T. castaneum*, or at the very least that the DCC genes of *msl2*, *msl3*, *mof*, and *mle* are not involved in male-specific dosage compensation. In contrast, the DCC of *T. castaneum* is composed of the non-specific lethal complex and plays a role in regulating a large number of critical genes related to the growth, development, and reproduction in both females and males [56]. Single-cell sequencing reveals that the escape from meiotic sex chromosome inactivation and postmeiotic reactivation of the X chromosome may be mediated by the dosage compensation machinery in *T. castaneum* [139].

### 3.4. The Dosage Compensation Mechanism of the Silkworm

*B. mori*, also known as the silkworm, is a lepidopteran model insect of great economic importance [140]. Several studies on WZ-bearing species indicate that dosage compensation is either lacking or incomplete in most lineages except for butterflies and moths, where the expression of the Z chromosome (chZ) in males (ZZ) is reduced by half to equal that in females (WZ). However, it is still unknown whether one chZ is inactivated (as in mammals) or both are partially repressed (as in *C. elegans*) [141]. Silkworms are organisms with female heterogamety (male ZZ, female WZ), and the main application of silkworms is the efficient production of silk proteins [141]. A Z-linked gene of the silkworm, named *T15.180a*, was firstly reported by Suzuki et al. in 1998, and the mRNA expression of this gene in males was twice as much as that in females [142]. The second gene, *Bmkettin*, located on the Z chromosome, also exhibited the same expression pattern as *T15.180a* [143]. In 2003, 13 genes surrounding the *Bmkettin* locus were identified, and most of these genes had higher mRNA expression levels in males compared to females [144]. Therefore, it is reasonable to speculate that the silkworms do not have dosage compensation [58]. Until 2011, studies found that the overall male/female expression ratio was not distinguishable from that of the Z chromosome to autosomes [145]. The total expression of the Z chromosome in males was significantly lower than that of the autosomes in males, which is an unexpected dosage compensation pattern [145].

Numerous analyses of experimental data reveal that the silkworm is likely to have a complete dosage compensation mechanism [145]. However, a recent study employing next-generation RNA sequencing technology to analyze the embryos and larval heads of silkworms at different stages found that dosage compensation occurred at 120 h in embryos, and additionally, the expression level of sex chromosomes in silkworms was lower than that of autosomes. Therefore, on the basis of these findings, the authors proposed that the dosage compensation in the silkworm is complete, yet it is different from the classical complete dosage compensation mechanism [79,146]. The homozygous sex chromosomes should have a transcriptional expression level that is nearly equal to that of the autosomes [79,146]. Another study discovered that somatic tissues of silkworms exhibited complete dosage compensation. However, it is not clear in gonadal tissue, and mixing gonad with somatic tissues may also obscure the identification of the dosage compensation types [80].

In the male silkworms, two copies of the Z chromosome were condensed and folded in a similar manner as revealed by the combination of fluorescence in situ hybridization (FISH) assay and genomics [141]. This finding supported the model of regulating Z-linked gene expression by partially or on average suppressing these two copies of the Z chromosome in males [141]. It also demonstrated that the suppression of the Z chromosome in males may emerge before the middle embryonic stage, which is earlier than previously thought [141]. Researchers recently visualized the autosomes and chZ chromosomes in somatic cells of both sexes by the Oligopaint DNA FISH assay, and they discovered that the chromosomes of *B. mori* are more compacted than those in *Drosophila*. This finding indicated that in the male silkworms, both chZs exhibit similarity in size and shape and are more compact than autosomes or the female chZ chromosome after DC establishment, suggesting that both male chZs are partially and equally down-regulated [141]. Another research revealed that a *Wolbachia* protein named Oscar can mainly target the Masculinizer (Masc) protein and this interaction thus leads to the male killing by a failure of dosage compensation in *B. mori* embryos [147]. There is a wide variety of research on the dosage compensation mechanism of silkworms. However, it remains controversial. The research methods vary, and so do the conclusions [148]. It is not difficult to envisage that for other lepidopteran insects, such as *M. sexta* [55], codling moth [149], *Plodia interpunctella* [81], and other species that have been researched on dosage compensation yet are seldom thoroughly studied, the dosage compensation mechanism theories may also be somewhat controversial.

### 3.5. The Dosage Compensation Mechanism of the Tobacco Hornworm

Complete dosage compensation was also discovered in *M. sexta* [55]. As another commonly used model organism in Lepidoptera, this insect is extensively employed in biomedical and biological science research. Gene expression in head tissues of males and females was acquired through high-throughput sequencing technology [55]. In general, dosage compensation was complete on the Z chromosome in both male and female tobacco hornworms. It seems likely that the dosage compensation in the *M. sexta* (WZ) species is achieved through the doubling of Z chromosome expression in females, rather than silencing one Z chromosome in males [55]. After filtering out the genes with extremely low expression levels, the average expression level of autosomal chromosomes was highly similar to that of the Z chromosome [55]. This indicated that for most genes in the tobacco hornworm, a complete dosage compensation effect exists [55]. Moreover, this compensation is associated with sex-specific gene expression and is essential for sexual dimorphisms [55].

## 4. Conclusions and Outlook

The dosage compensation mechanism compensates for the disparities in gene expression resulting from sex differences in organisms and plays an essential role in bisexual eukaryotes. Researchers have extensively conducted investigations into the dosage compensation effects in three phylogenetically distant model organisms, namely, *C. elegans*, *Drosophila*, and humans. Although diverse mechanisms evolve to perform dose compensation across species, the core mechanism is accomplished through the regulation of the X-linked gene expressions. To achieve dose compensation, the nematodes halve expression of both X chromosomes in females. In *Drosophila*, the X chromosome expression is doubled only in males. In contrast, one of the two X chromosomes is inactivated in mammalian females. Understanding the dosage compensation mechanisms across species can provide fundamental insights into the evolutionary conservation and divergence.

Dosage compensation is of crucial importance for the sex differentiation of insects. There exist certain trends in the patterns and mechanisms underlying dosage compensation in insects. However, a strictly universal principle has yet to be established. In some insect species, the regulation of dosage compensation is chromosome-wide. On the contrary, dosage compensation exhibits a more gene-specific trait in other insects rather than affecting the entire chromosome. In insect species with heteromorphic sex chromosomes, the expression of sex-linked genes is at least partially equalized between the two sexes. Moreover, the ultimate objective of dosage compensation in insects and other organisms shares a common characteristic, which is to balance the expression of sex-linked genes between different sexes, thereby ensuring the normal growth and development of organisms. Nevertheless, the mechanisms and regulatory molecules of dosage compensation differ among insect species.

Although certain key genes associated with insect sex determination have been identified, the manner in which these genes collaborate as well as their upstream and downstream regulators remain unclear. Therefore, it is necessary to conduct in-depth studies on the functions and interrelationships of these genes. Subsequently, a more comprehensive regulatory network for sex determination should be constructed. Given the limited number of known dosage compensation patterns and mechanisms, additional investigations are essential to fully reveal their molecular diversity and to identify whether certain mechanisms are more widespread or have undergone repeated evolution. Environmental factors also have an impact on insect sex determination and sometimes interact with the genetic factors. Further research on how environmental factors influence the sex determination gene expressions and dosage compensation will strengthen our understanding of how insects maintain the balance of sex ratios under diverse environmental conditions.

Differences also exist in the mechanisms of dosage compensation between male heterogamety (XX/XY) and female heterogamety (WZ/ZZ). For example, in the process of XY-type sex determination, fruit flies employ the *Sxl* gene as a key switch. This process is mediated by the MSL complex, which is responsible for dosage compensation by doubling the expression of the X chromosome specifically in males. In contrast, silkworms utilize *Feminizer* (*Fem*) gene as the upstream regulators for WZ-type sex determination. This regulation is mediated by the Masc protein, which plays a role in dosage compensation by suppressing the expression of both male Z chromosomes. However, in both the fruit flies and silkworms, the terminal genes within the sex determination cascade are the constant *doublesex*, which encode male- and female-specific protein products and govern the expression of downstream sex-related genes.

To better elucidate the mechanism of dosage compensation and gene regulation in insects, we can utilize gene editing technology to precisely alter the sex of insects and guide their sex determination to develop in a direction beneficial to humans. In this case, we can also employ the strategy of sex determination and differentiation to control the pest population and expand the scale of natural enemies, for instance, by obtaining sterilized male pests by the radiation sterility method and subsequently releasing them into the wild to mate with other normal females, thereby achieving the goal of controlling the pest population. Furthermore, for some resource insects with economic importance, such as the silkworm, research on its sex determination mechanism can provide technical support for sex control in sericulture of males.

For the evolution from *Drosophila* to humans, dosage compensation has evolved various mechanisms, characterized by the diversity of its components. *D*. melanogaster is the representative insect of Diptera, the dosage compensation mechanism of which is thoroughly understood. *Sciara ocellaris* is another Dipteran insect with an X0 sex determination system: females are XX and males are X0. However, homologous proteins between *Drosophila* and *Sciara* perform different functions. For example, the *Sxl* gene of *Sciara* has no discriminative function in governing sex determination and dosage compensation. Additionally, the *doublesex* gene of *Sciara* is implicated in sex determination but lacks the discriminatory role. In comparison with *Drosophila*, there exist homologous proteins of MLE, whereas MSL2 is absent in *Sciara*. Thus, investigating the functional differences between species is of great significance for understanding the evolution mechanism and environmental adaptation of species.

In recent years, high-throughput sequencing technologies have become increasingly prominent in research on dosage compensation mechanisms. Single-cell sequencing can be employed for transcriptome analysis of insect cells of different genders and developmental stages. This novel approach inevitably contributes to illustrating the cell heterogeneity and dynamic changes in gene expression during the processes of sex determination and dose compensation. However, except for *Drosophila*, the sex determination and dosage compensation mechanisms in other insects remain unclear. The mechanism in Lepidoptera is even controversial, which also demonstrates the complexity and diversity in dosage compensation processes. Additionally, the mechanisms of various insects also show remarkable differences. In the future, through the integration of multi-omics joint analysis, the expression profiles, protein interaction networks, and epigenetic modifications of insects with different genders will be fully utilized for comprehensively uncovering the mechanism of gender balance and the evolution of dosage compensation in insects.

## Data Availability

Data contained within the article.
